# Phospholipase C inhibits apoptosis of porcine primary granulosa cells cultured in vitro

**DOI:** 10.1186/s13048-019-0567-4

**Published:** 2019-09-25

**Authors:** Huali Chen, Youfu Yang, Youlin Wang, Yuan Li, Yamei He, Jiaxin Duan, Dejun Xu, Yifei Pei, Jianyong Cheng, Li Yang, Rongmao Hua, Xiaoya Li, Jie Wang, Xiaohan Jiang, Huanshan He, Lin Wu, Dingbang Liu, Qingwang Li

**Affiliations:** 1 College of Animal Science and Technology, Northwest A&F University, Yangling, Shaanxi 712100 People’s Republic of China; 2 Hanzhong Vocational and Technical College, Hanzhong, 723000 Shaanxi China; 3 First Affiliated Hospital of Jiamusi University, Jiamusi, 154007 Heilongjiang China

**Keywords:** Phospholipase C, Apoptosis, Granulosa cells, Porcine

## Abstract

Phospholipase C (PLC) can participate in cell proliferation, differentiation and aging. However, whether it has a function in apoptosis in porcine primary granulosa cells is largely uncertain. The objective of this study was to examine the effects of PLC on apoptosis of porcine primary granulosa cells cultured in vitro. The mRNA expression of *BAK*, *BAX* and *CASP3*, were upregulated in the cells treated with U73122 (the PLC inhibitor). The abundance of *BCL2* mRNA, was upregulated, while *BAX* and *CASP3* mRNA expression was decreased after treatment with m-3M3FBS (the PLC activator). Both the early and late apoptosis rate were maximized with 0.5 μM U73122 for 4 h. The rate of early apoptosis was the highest at 4 h and the rate of late apoptosis was the highest at 12 h in the m-3M3FBS group. The protein abundance of PLCβ1, protein kinase C β (PKCβ), calmodulin-dependent protein kinaseII α (CAMKIIα) and calcineurinA (CalnA) were decreased by U73122, and CAMKIIα protein abundance was increased by m-3M3FBS. The mRNA expression of several downstream genes (CDC42, NFATc1, and NFκB) was upregulated by PLC. Our results demonstrated that apoptosis can be inhibited by altering PLC signaling in porcine primary granulosa cells cultured in vitro, and several calcium^−^sensitive targets and several downstream genes might take part in the processes.

## Introduction

In mammalian ovaries, granulosa cells have been demonstrated to play a critical role in deciding the destiny of follicles, follicular growth and maintenance as well as their apoptotic process [1]. More than 99% of follicles degenerate and granulosa cell apoptosis play an important role in the process of follicular atresia [2]. Granulosa cells apoptosis appears to be an integral part of follicle development, and reflects the mitogenic growth of the follicle [3]. In addition, an effector caspase (such as *caspase-3*) could be activated by an initiator to enforce apoptotic cell death [2, 4], which could also be activated by *P53.* While *P53* activates *BAX* and is protected by the regulation of *BCL2* [4].The relative expression levels of pro-apoptotic and anti-apoptotic factors in granulosa cells determine whether an ovarian follicle will grow or experience atresia in the late preantral stage and affect oocyte ovulation [5–7].

Phospholipases can be found in several different organisms, including bacteria, animals, and viruses [8]. Phospholipase C (PLC) is a key enzyme in phosphoinositide metabolism that performs cell proliferation/differentiation, the secretion of hormones, fertilization, cell motility and other functions [9, 10]. PLCβ1, the most extensively investigated PLC isoform, is a critical factor in the regulation of nuclear inositol lipid signaling [11]. PLCβ plays an important role in the Wnt/Ca^2+^ pathway, which promotes the release of intracellular Ca^2+^ and affects Ca^2+^ sensitive targets, containing protein kinase C (PKC), Ca^2+^-calmodulin-dependent protein kinaseII (CAMKII) and Ca^2+^-calmodulin-sensitive protein phosphatase calcineurin (Caln) [12, 13]. Both CAMKII and PKC activate NFκB, and Caln activates cytoplasmic protein nuclear factor associated with T cells (NFAT) via dephosphorylation [14, 15].

The activations of PLC and PKC can play a role in the physiological cumulus expansion before ovulation in mouse [16], and involve in mouse embryonic stem-cell proliferation and apoptosis [17]. But there are little reports about the role of PLC on apoptosis of porcine granulosa cells. Given the pivotal role of granulosa cells apoptosis in follicular development and atresia [1, 18], we set out to determine whether apoptosis could be regulated by PLC in porcine granulosa cells and how the Ca^2+^, several Ca^2+^ sensitive proteins and downstream genes could be changed, using the in vitro primary granulosa cells as a model system.

## Methods

The animal use protocol was approved by the Institutional Animal Care and Use Committee of the College of Animal Science and Technology, Northwest A&F University, Yang Ling, China.

### Preparation of the porcine granulosa cells

The pigs for the experiment were from a local slaughter house. They were a cross of A × (B × C), in which A was the terminal male Duroc, B was the matriarchal father Landrace, and C was the matriarchal mother Yorkshire. All of the pigs were 6–7 months old and weighed approximately 115 kg. Porcine ovaries were collected and washed as described [19]. Follicular fluid was harvested by aseptic aspiration with a 26 gauge needle [20] from medium-sized (3–5 mm indiameter) healthy follicles, and porcine granulosa cells were prepared as described [19].

### Culture of the granulosa cells

All reagents and chemicals were obtained from Solarbio Life Sciences (Solarbio, Beijing, China) unless otherwise stated. The porcine granulosa cells were incubated in a basic medium consisting of DMEM/F12 (Gibco, California, USA) with 0.3% bovine serum albumin (BSA) (Roche; Basel, Switzerland), 3% fetal bovine serum(Serapro, Systech Gmbh, Germany), 5 ng/ml sodium selenite, 10 mmol/L NaHCO_3_, a nonessential amino acid, 50 ng/mL insulin, 0.1 IU/mL FSH, and 1% antibiotics. This medium was used as a control, and the cells were at a density of 1 × 10^6^/mL and incubated in a humidified incubator at 37 °C with 5% CO _2_ for 36–44 h before changing to a serum-free culture with 2.5 μg/ml transferrin for 24 h. Then half of the medium (500 μl) was exchanged with fresh solution every 24 h as the experiment required; several doses of U73122 (the PLC inhibitor) in DMF or m-3M3FBS (the PLC activator) in DMSO were added into the culture with final concentration of 0 μM (control), 0.05 μM, 0.5 μM, 5 μM, 50 μM. Expression of genes other than PLCB1, all proteins and intracellular Ca^2+^ concentration were assessed at 4 h posted treatment, whereas expression of PLCB1 gene and cell apoptosis which was analyzed using flow cytometry were evaluated at 2, 4, 8, 12, 24 and 48 h after incubation.

### Immunofluorescence

Follicle-stimulating hormone receptor (FSHR), which is mainly located in the cytoplasm and appears red under the fluorescent microscope after immunostaining, is the marker protein of porcine granulosa cells. In addition, DAPI dye can penetrate cell membranes and produce fluorescence by binding to nucleic acids in the nucleus, making the cells appear blue under the microscope.

After the cells grew on the glass slide for 36–44 h, they were fixed in 4% paraformaldehyde for 30 min and washed three times with PBS. The porcine granulosa cells were permeabilized with 0.2% Triton X-100 and then blocked using 10% normal serum in 1% BSA in TBS (10 mM tris-HCl, 150 mM NaCl, and 0.1% Tween 20; pH 7.5) for 1 h at room temperature. The cells were incubated with anti-FSHR antibodies (Bioss, Beijing, China; Cat No.: bs-0895R, diluted 1:350) in 1% BSA in TBS overnight at 4 °C. The slides with cells were washed twice for 5 min each time, followed by incubation with Cy3 conjugated Goat Anti-rabbit IgG (H + L) (Servicebio, Wuhan, China) (Cat No.: GB21303, 1:300) for 60 min. The nuclei were identified by 4,6-diamidino-2-phenylindole (DAPI) staining. Nonimmune rabbit IgG was used as the negative control. The slides were imaged using a Nikon Eclipse Ti microscope (Nikon, Tokyo, Japan). The data were analyzed with Image J software (National Institutes of Health, Bethesda, MD, USA).

### RNA extraction and RT-qPCR

The granulosa cells were treated for 2 h, 4 h, 8 h, 12 h, 24 h and 48 h which was used for detecting PLCB1 mRNA expression or for 4 h which was used for detecting other genes, and then total RNA was extracted from the cells using TRIzol reagent (Takara, Kyoto, Japan) as described [21]. RNA concentration was determined using a spectrophotometer (NanoDrop 2000c, Thermo Scientific, Waltham, Massachusetts, USA) by absorbance at 260 nm [22]. Then, 1 μg of total RNA was converted to cDNA using 5 × All-In-One RT MasterMix (Abm, Vancouver, Canada) according to the manufacturer’s protocol [22]. Primers were synthesized by Sangon Biotech (Shanghai, China), and the primer sequences used for genes studied are listed in Table 1. The RT-qPCR reactions were performed in triplicate using the EvaGreen 2 × qPCR MasterMix-no Dye (Abm, Vancouver, Canada) with a Bio-Rad CFX96 system (BioRad, California, USA) according to the manufacturer’s protocol. The mRNA relative expression of each gene was calculated comparing to the housekeeping gene β-actin. The final relative expression fold differences, with gene expression NC as a control, were calculated with the 2 ^− ∆∆Ct^ method for each gene [19].

**Table 1:** Primer Sequences used in this study

Primer	Primer sequence (5′-3′)	Genebank ID	Product size/bp
β-Actin	F-5′- ATCAAGATCATCGCGCCTCC-3’	XM_003124280.4	169
	R-5′- AATGCAACTAACAGTCCGCCT-3’		
PLCB1	F-5′-TGAGAAGGAGGGCAGCTTTGGA-3’	XM_013985062.1	108
	R-5′-CAGCGAGGTCTTGCTCAATGGT-3’		
Cdc42	F-5′- ATGTGGAGTGTTCTGCACTCA-3’	XM_005656039.2	132
	R-5′-GGCTCTGGAGAGACGTTCAT −3’		
NFATC1	F-5′-GAAAACCGACGGAGACCTGT-3’	NM_214161.1	172
	R-5′-TATGACTGGAGCGTTGGCAG-3’		
NFATC2	F-5′-TCCTACCCCACGGTCATTCA-3’	NM_001113452.1	179
	R-5′-TGTATCCAGCTAAGGTGTGTGTC −3’		
NFKB	F-5′-GAGGTGCATCTGACGTATTC − 3’	NM_001048232.1	138
	R-5′- CACATCTCCTGTCACTGCAT-3’		
NLK	F-5′-GCGGCTTACAATGGCGGTACAT-3’	XM_013981185.1	122
	R-5′-TGAAGATGGTGCTGAGGGTGGT-3’		
CTNNB1	F-5′-TCGCCTTCACTACGGACTACCA-3’	NM_214367.1;XM_013981492.1; XM_005669377.2;XM_013981494.1	180
	R-5′-TGATGAGCACGAACCAGCAACT-3’		
BAK1	F-5′-GACAGAAGTGGGGCAAGATCA-3’	XM_001928147.3	190
	R-5′-CGCTCCAATCAGCTCCCTCT-3’		
BAX	F-5′-GGCCTCCTCTCCTACTTTGG −3’	XM_013998624.2; XM_003127290.5	103
	R-5′-CTCAGCCCATCTTCTTCCAG −3’		
BCL2	F-5′-ATCGCCCTGTGGATGACTGAGT-3’	XM_003121700.4	140
	R-5′-GCCTTCAGAGACAGCCAGGAGA-3’		
CASP3	F-5′-ATACACGTACTCATGGCGAAC-3’	XM_005671704.2	102
	R-5′-TCCAGAGTCCACTGATTTGCT-3’		
CASP8	F-5′-CCAGGATTTGCCTCCGGTTA −3’	NM_001031779.2	144
	R-5′-TGTGGGATGTAGTCCAGGCT −3’		
TP53/P53	F-5′-TCGGCTCTGACTGTACCACCAT −3’	NM_213824.3	155
	R-5′-GGCACAAACACGCACCTCAAAG-3’		

“F” represented forward primer, and “R” represented reverse primer

### Western blotting

Western Blotting was measured as described previously [19] after treatment for 4 h. The protein concentrations were determined by BCA protein assay (Beyotime, Hangzhou, China), and the proteins used were 35 μg (for PLCB1) or 15 μg (for others). The primary antibodies were PLCB1 (Ommi mAbs, USA; OM116324,1:1000 dilution), PKC beta1 + 2 (Bioss, Beijing, China; bs-0267R, 1:2000 dilution), CAMKIIα (WanleiBio, Shen Yang, China; WL03453, 1:1200 dilution), calcineurin A (WanleiBio, Shen Yang, China; WL03449,1:600 dilution) and β-actin (Santa Cruz Biotechnology, CA, USA; SC-1615, 1:500 dilution).The secondary antibodies were Goat anti-Rabbit lgG(H + L)-HRP (Sungene Biotech, Tian Jin, China; LK2001, 1:4000 dilution) for PLCB1, PKC beta1 + 2, CAMKIIα and calcineurin A or Goat anti-Mouse lgG(H + L)-HRP (Sungene Biotech, Tian Jin, China; LK2003, 1:4000 dilution) for β-actin. The membranes were visualized using ECL (CW Bio, Beijing, China). The data were analyzed with Quantity One (BioRad, California, USA).

### Flow cytometry analysis of the cell apoptosis

The cells were seeded into 6-well culture plates at a density of 1 × 10 ^6^/mL cells per well. The cells were treated with 0.5 μM U73122 or m3M3FBS for 2 h, 4 h, 8 h, 12 h, 24 h, and 48 h, respectively, and then the cells were washed three times with cold PBS by centrifugation at 800 g for 5 min. Cell apoptosis was assessed with an annexin V-FITC/propidium iodide apoptosis detection kit (Vazyme Biotech, Nanjing, China) according to the manufacturer’s instructions, with the cell pellet resuspended in 100 μL of 1 × binding buffer and adjusted to the density to 1 × 10 ^6^/mL. The stain was detected within 1 h by a flow cytometry instrument (Beckman Coulter, CA, USA).

### Determination of intracellular Ca^2+^ concentration

The cells at a density of 8 × 10 ^5^/mL cells per well were treated with 0.5 μM U73122 or m3M3FBS for 4 h. The concentration of intracellular Ca^2+^ was monitored by a fluorescent Ca^2+^ indicator Fluo 3-AM (Sigma, St. Louis, MO, USA), and was measured as described previously [23]. The intracellular Ca^2+^ changes were measured with the ratio of fluorescence intensities excited at 488 nm and 530 nm using flow cytometry (CyFlow Cube, PARTEC, Germany).

### Statistical analysis

All experiments were carried out at least three times.GraphPad Prism 6.0 was used to graph the results. The data were analyzed using SPSS 19.0 software (SPSS science, Chicago, IL, USA), multiple comparisons among the groups were evaluated by Duncan’s test. One-way ANOVA that conforms to normal distribution and homogeneity of variance was used to determine differences among multiple groups which was marked by letters, and an independent sample *t*-test that conforms to normal distribution was used to determine the difference between two groups which was marked by asterisk. The data are presented as the means ± SEM. A value of *p* < 0.05 was considered statistically significant. For each treatment, the means without common letters are significantly different (*p* < 0.05), and means with * indicate a difference at *p* < 0.05, means with ** indicate a difference at *p* < 0.01, and means with *** indicate a difference at *p* < 0.001. The data of cell apoptosis and intracellular Ca^2+^ concentration were analyzed with FlowJo V10 (Leonard Herzenberg’s laboratory, Stanford University, USA).

## Results

### The identification of porcine granulosa cells

**Fig. 1:**
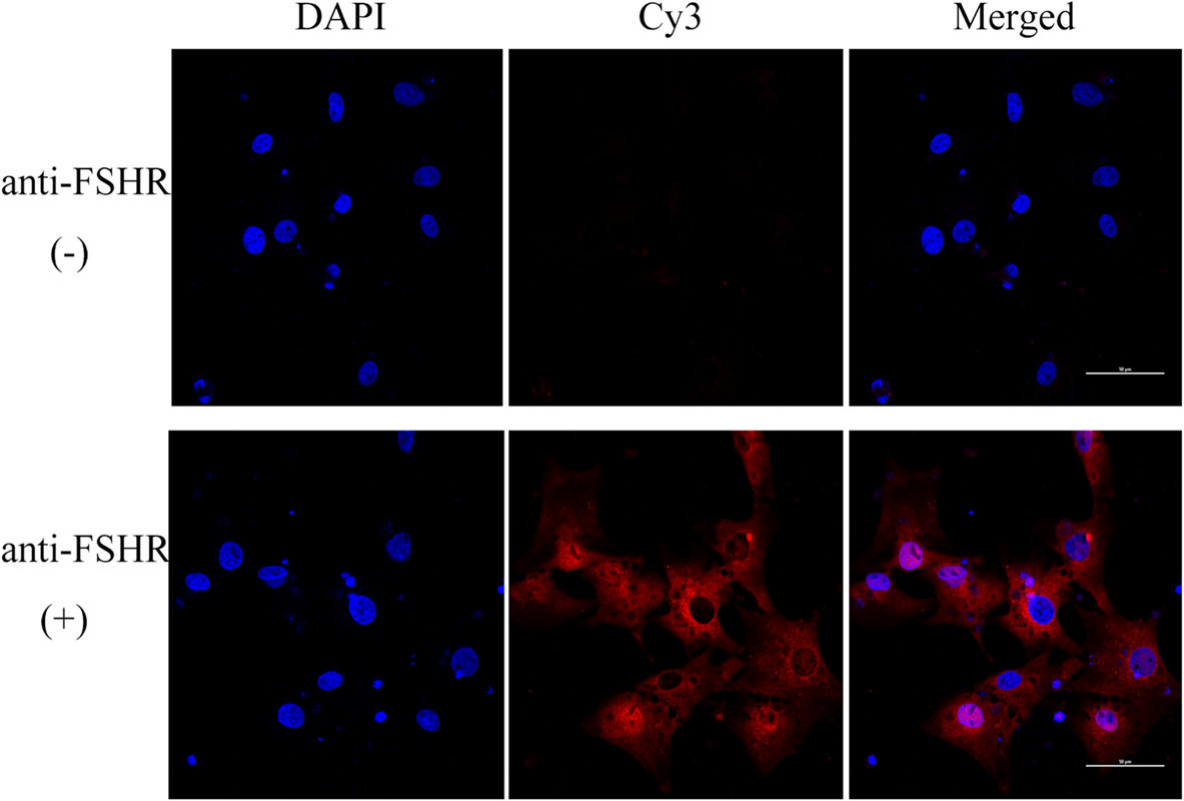
The identification of porcine granulosa cells by immunofluorescence. Porcine granulosa cells were stained with FSHR; The cell nuclei in the entire visual field were stained by DAPI; Most of the DAPI could be merged with FSHR. Magnification, × 600; Scale bar = 50 μM

As porcine granulosa cells were the primary cell types, it was necessary to identify them by a specific marker. FSHR was expressed specifically in the porcine granulosa cells in an immunofluorescence analysis, and the nuclei in the entire visual field were stained by DAPI (Fig. 1). The results of immunofluorescence showed that the rate of FSHR-positive cells was more than 98%, which indicated that the purity of the porcine granulosa cells isolated from porcine ovaries was high enough for them to be used subsequently.

### The influence of PLC inhibitor or activator on PLC expression in porcine granulosa cells in vitro

The *PLCB1* mRNA abundance supplied with 0.5 μM U73122 was the lowest (*p* < 0.05) in porcine granulosa cells after treatment for 2 h, 4 h, 8 h, 12 h and 24 h in porcine granulosa cells, and did not change after treatment for 48 h (Additional file 1: Figure S1A-E). The mRNA expression of *PLCB1* in the porcine granulosa cells was the highest in the 0.5 μM concentration group among all the concentration groups before 12 h (*p* < 0.05; Additional file 1: Figure S2A-C), and first increased in the 0.05 μM concentration group (*p* < 0.05) and then decreased when the concentration reached 5 μM (*p* < 0.05) of m-3M3FBS before 48 h (Additional file 1: Figure S2D and S4E). There was no difference at 48 h among the different concentrations of U73122 or m-3M3FBS (*p* > 0.05; Additional file 1: Figure S1F and S2F).

**Fig. 2:**
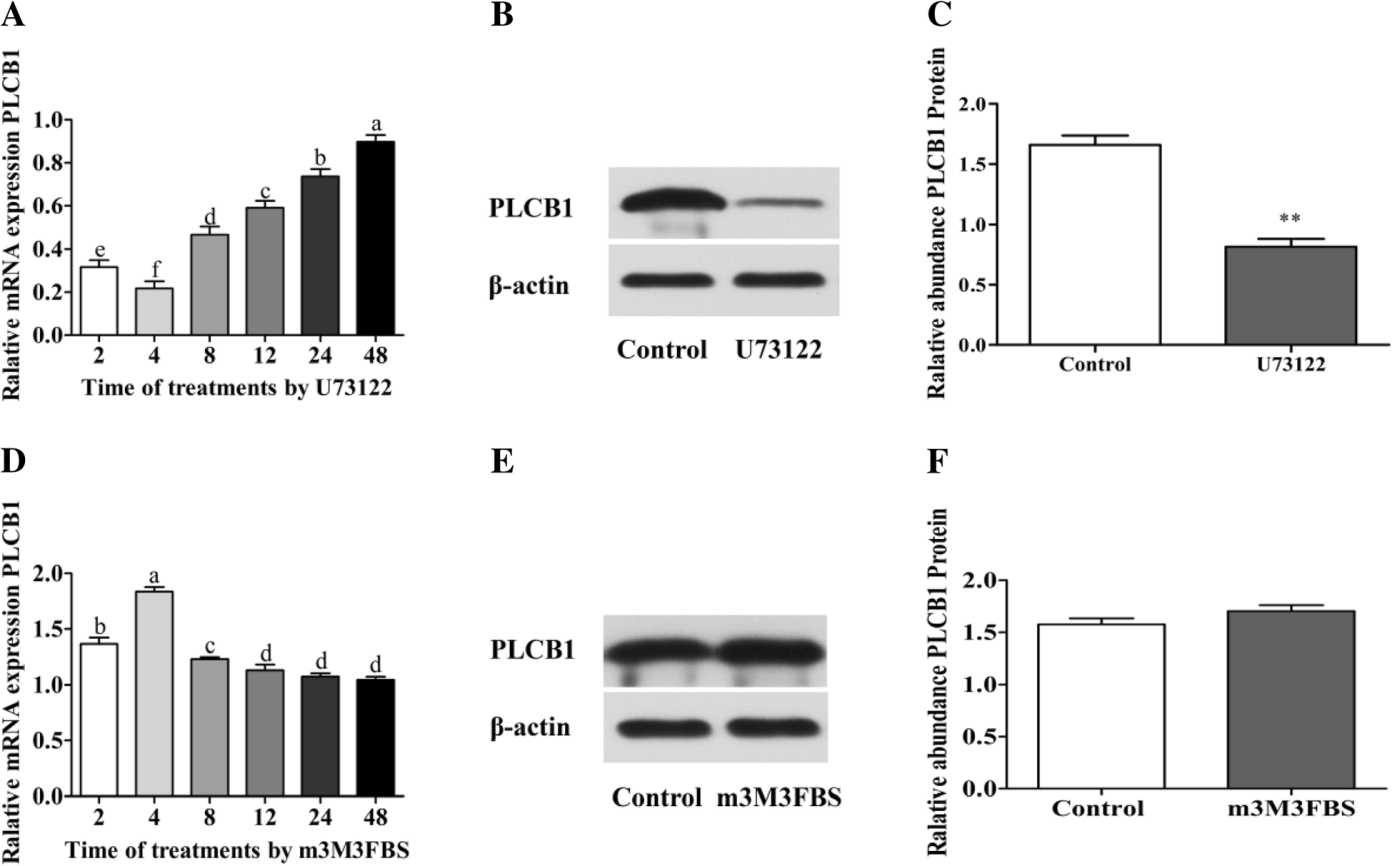
The influence of PLC inhibitor and activator on PLC mRNA and protein expression in porcine granulosa cells in vitro**.** Data are mean ± S.E.M.of three independent replicates. (**A**) The PLCB1 mRNA expression treated with 0.5 μM U73122 for the times given. (**B**) One representative western blot band chart for PLCB1 treated with 0.5 μM U73122 for 4 h. (**C**) The PLCB1 protein abundance treated with 0.5 μM U73122 for 4 h. (**D**) The PLCB1 mRNA expression treated with 0.5 μM m3M3FBS for the times given. (**E**) One representative western blot band chart for PLCB1 treated with 0.5 μM m3M3FBS for 4 h. (**F**) The PLCB1 protein abundance treated with 0.5 μM m3M3FBS for 4 h. One-way ANOVA was used for genes and independent sample *t*-test was used for proteins. For each treatment, means without common letters are significantly different (*p*<0.05) (**A** and **D**); means with ** indicate a difference at *p*<0.01 compared with control (**C**); means without asterisk are not significantly different (**F**)

The most effective concentration of U73122 or m3M3FBS was 0.5 μM overall, except at 48 h. The *PLCB1* mRNA abundance of the different time points (2 h, 4 h, 8 h, 12 h, 24 h and 48 h) of the porcine granulosa cells treated with 0.5 μM U73122 or m3M3FBS was compared. The mRNA expression of *PLCB1* was the lowest at 4 h in porcine granulosa cells cultured with 0.5 μM U73122 (*p* < 0.05) and greatly increased after 4 h, resulting in a difference among the time gradients (*p* < 0.05; Fig. 2A). Compared to the control group, the relative abundance of the PLCB1 protein was decreased with the addition of 0.5 μM U73122 for 4 h (*P* = 0.001; Fig. 2B and C). The *PLCB1* mRNA abundance in the porcine granulosa cells was the highest with 0.5 μM m-3M3FBS at 4 h (*p* < 0.05), and was greatly decreased at 8 h and 12 h (*p* < 0.05), but there was no difference after 12 h (Fig. 2D). The increase in PLCB1 protein abundance did not change in the m-3M3FBS treatment group (*p* > 0.05; Fig. 2E and F).

### The influence of PLC inhibitor and activator on apoptosis of porcine granulosa cells in vitro

**Fig. 3:**
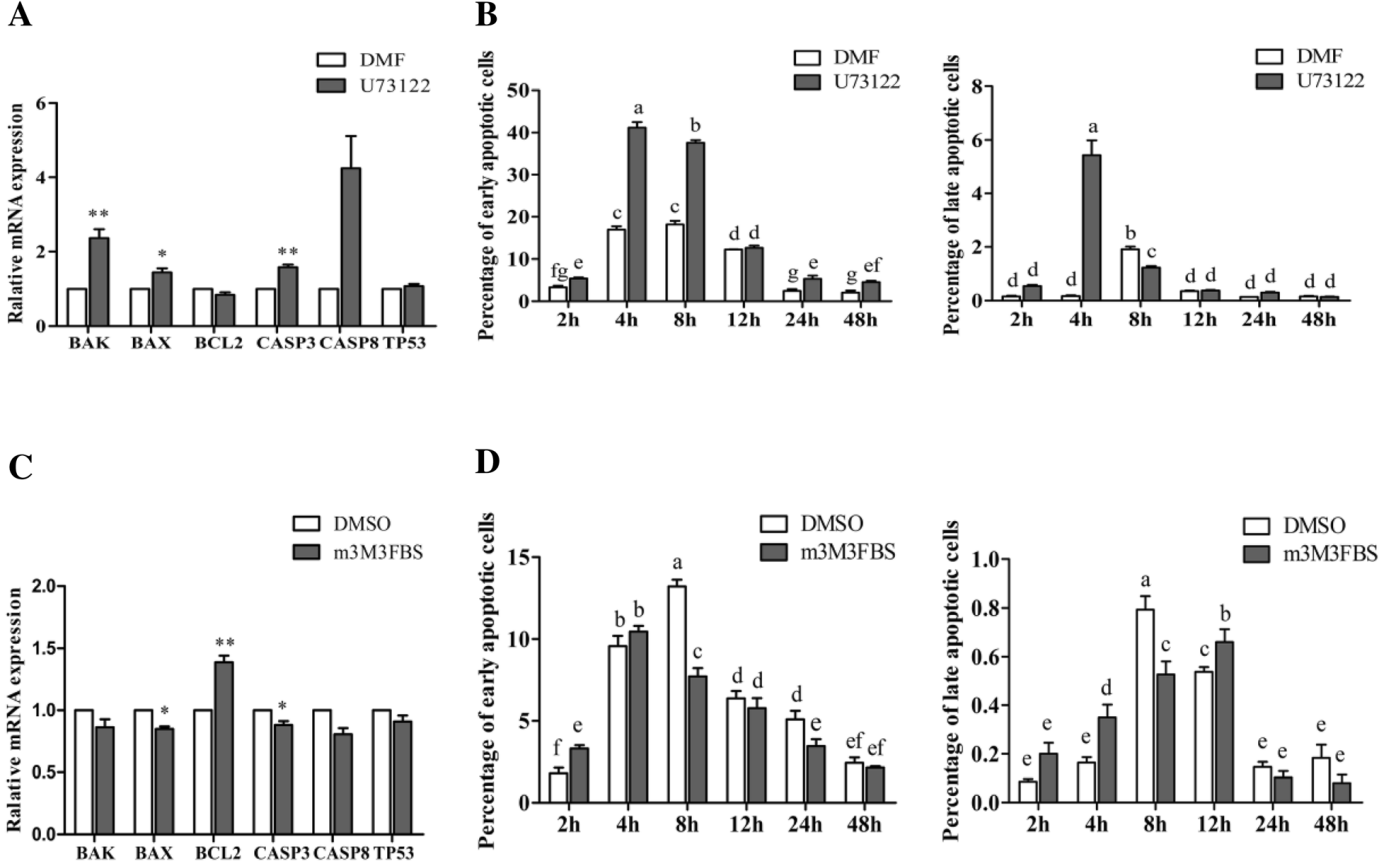
The influence of PLC inhibitor and activator on apoptosis of porcine granulosa cells in vitro. The cells were challenged with 0.5 μM U73122 or m-3M3FBS for 4 h (genes) or for the times given (percentage of apoptotic cells), which were processed for annexin V/PI staining and measured by flow cytometry assay. (**A**) The effect of U73122 on the abundance of the mRNAs that encode the transcription factors involved in the regulation of apoptosis of porcine granulosa cells. (**B**) The percentage of early apoptosis and late apoptosis treated with 0.5 μM U73122 at different times. (**C**) The effect of m3M3FBS on the abundance of the mRNAs that encode the transcription factors involved in the regulation of apoptosis of porcine granulosa cells. (**D**) The percentage of early apoptosis and late apoptosis treated with 0.5 μM m-3M3FBS at different times. Data are mean ± S.E.M. of three independent replicates. Independent sample *t*-test was used for (**A**) and (**C**), and one-way ANOVA was used for (**B**) and (**D**). For each treatment, means without asterisk are not significantly different, means with * indicate a difference at *p* <0.05 compared with control, means with ** indicate a difference at *p* <0.01 compared with control (**A** and **C**); or means without common letters are significantly different(*p*<0.05) (**B** and **D**)

The effect of U73122 or m3M3FBS on the expression of the mRNA of the factors involved in the regulation of apoptosis of porcine granulosa cells was measured. The mRNA abundance of *BAK* (*p* < 0.01), *BAX* (*p* < 0.05) and *CASP3* (*p* = 0.001) was upregulated, while there was no effect on the mRNA expression of *TP53*, *CASP8* and *BCL2* with the addition of 0.5 μM U73122 for 4 h in porcine granulosa cells (Fig. 3A). The mRNA abundance of *BCL2* was upregulated (*p* < 0.01), and the mRNA expression of *BAX* and *CASP3* was downregulated (*p* < 0.05) after treatment with 0.5 μM m-3M3FBS for 4 h, while *BAK*, *CASP8* and *TP53* mRNA abundance did not change (*p* > 0.05; Fig. 3C).

The apoptois of granulosa cells treated with the inhibitor U73122 or the activator m-3M3FBS for 2 h, 4 h, 8 h, 12 h, 24 h and 48 h were measured by a flow cytometry assay, with Q1(FITC-PE+), Q2(FITC+PE+), Q3(FITC+PE-), and Q4(FITC-PE-) representing the cell debris, late apoptosis, early apoptosis and the normal cells, respectively (Additional file 1: Figure S3 and S4). Apoptosis increased at first and then decreased as the treatment time increased treated by U73122 or m-3M3FBS, but the apoptosis trends were different in the two groups (Additional file 1: Figure S3 and S4). The rates of early apoptosis and late apoptosis of the cells treated by U73122 for 4 h were the highest among all time points (*p* < 0.05), then gradually decreased at 8 h and 12 h time point (*p* < 0.05), and remained a steady level after 24 h (*p* > 0.05; Fig. 3B). The rates of early apoptosis and late apoptosis in the m-3M3FBS supplement group were the highest in the 4 h and 12 h treatment groups (*p* < 0.05) and then decreased (*p* < 0.05) with the treatment time increased except at 48 h (*p* > 0.05; Fig. 3D).

### The effect of PLC inhibitor and activator on intracellular Ca^2+^ in porcine granulose cells

**Fig. 4:**
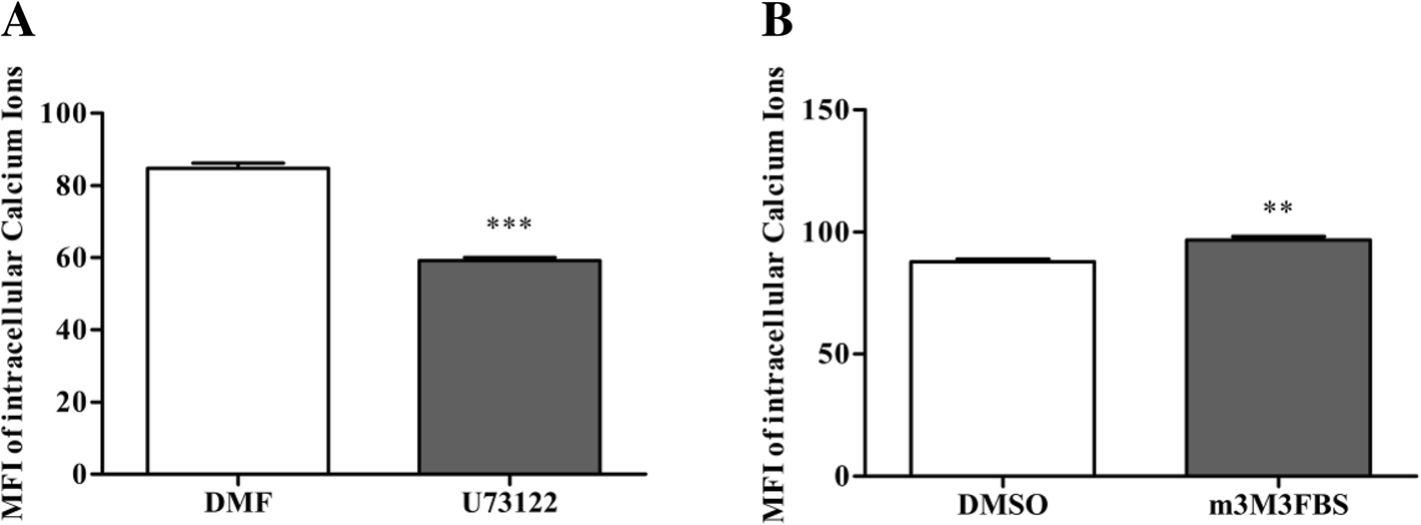
The effect of U73122 and m3M3FBS on intracellular Ca^2+^ in porcine granulose cells.The cells were challenged with 0.5 μM U73122 or m3M3FBS for 4 h. **a** Median fluorescence intensity of intracellular Ca^2+^ treated with U73122. **b** Median fluorescence intensity of intracellular Ca^2+^ treated with m-3M3FBS. Data are mean ± S.E.M. of three independent replicates. Independent sample *t*-test was used. For each treatment, means with ** indicate a difference at *p*<0.01 compared with control (**b**), and means with *** indicate a difference at *p*<0.001 compared with control (**a**)

Flow cytometry was used to monitor the variation of intracellular Ca^2+^ concentration (Additional file 1: Figure S5), and the values were represented by median fluorescence intensity (MFI), which was calculated using FlowJo (V10). Compared with the control group, intracellular Ca^2+^ concentration with the addition of 0.5 μM U73122 for 4 h was declined (*p* < 0.001; Fig. 4a), and the intracellular Ca^2+^ concentration was increased (*p* < 0.01) cultured with 0.5 μM m-3M3FBS for 4 h in porcine granulosa cells (Fig. 4b).

### The influence of PLC inhibitor or activator on the protein abundance of three Ca^2+^-sensitive proteins and the mRNA expression of downstream genes and CTNNB in porcine granulosa cells in vitro

**Fig. 5:**
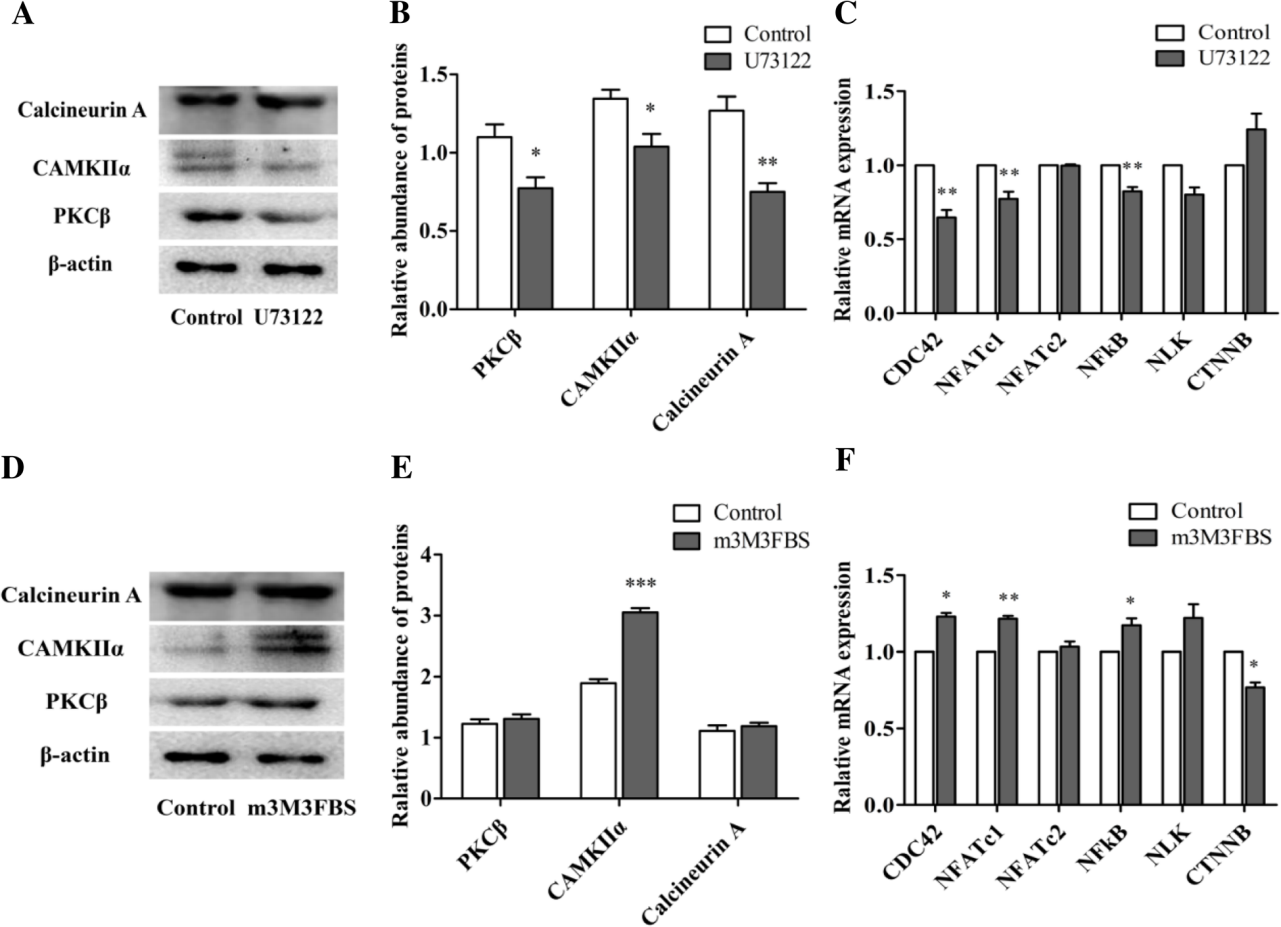
The effect of U73122 and m-3M3FBS on Ca^2+^ sensitive proteins (PKCβ, CAMKIIα and CalcineurinA protein) and mRNA expression of downstream genes and CTNNB in porcine granulosa cells**.** The cells were challenged with 0.5 μM U73122 or m-3M3FBS for 4 h. **a** One representative western blot band chart for PKCβ, CAMKIIα and calcineurinA treated with U73122. **b**The protein abundance of PKCβ, CAMKIIα and calcineurinA treated with U73122. **c** The effect of U73122 on mRNA expression of downstream genes and CTNNB. **d** One representative western blot band chart for PKCβ, CAMKIIα and calcineurinA treated by m3M3FBS. **e** The protein abundance of PKCβ, CAMKIIα and calcineurinA added by m-3M3FBS. **f** The effect of m3M3FBS on mRNA expression of downstream genes and CTNNB. Data are mean ± S.E.M. of three independent replicates. Independent sample *t*-test was used. For each treatment, means without asterisk are not significantly different, means with * indicate a difference at *p*<0.05 compared with control, means with ** indicate a difference at *p*<0.01 compared with control, and means with *** indicate a difference at *p*<0.001 compared with control

Compared to the control group, the PKCβ (*p* < 0.05), CAMKIIα (*p* < 0.05) and Caln A (*p* < 0.01) protein abundance was decreased with the addition of 0.5 μM U73122 for 4 h (Fig. 5a and b). The mRNA expression of *CDC42*, *NFATc1* and *NFkB* was decreased after treatment with 0.5 μM U73122 for 4 h (*p* < 0.01), but the *NFATc2, NLK* and CTNNB mRNA abundance was not changed (*p* > 0.05; Fig. 5c). The abundance of the CAMKIIα protein was increased (*p* < 0.001), but the expression of PKCβ and calcineurin A did not change treated with m-3M3FBS (Fig. 5d and e). The mRNA expression of *CDC42* (*p* = 0.01), *NFATc1* (*p* < 0.01), and *NFkB* (*p* < 0.05) was increased with the addition of 0.5 μM m-3M3FBS, but *NFATc2* and *NLK* mRNA abundance did not change (*p* > 0.05; Fig. 5f). However, the expression of *CTNNB* mRNA was reduced with the addition of 0.5 μM m-3M3FBS for 4 h (*p* < 0.05; Fig. 5f).

## Discussion

PLCs are important enzymes that is thought to play a role in the physiological regulation of organisms [9, 10], and could participate in the apoptosis of mouse embryo stem-cells [17].Whether PLC play a part in apoptosis of porcine granulose cells was unclear [24]. U73122 is an inhibitor of the PLC protein widely used in mammals [25, 26], and m-3M3FBS is an activator of PLC protein which could participate in several physiology regulation [27, 28]. In this study, the influence of PLC inhibitor and activator on PLC mRNA and protein expression, apoptosis, intracellular Ca^2+^ concentration and several targeted proteins and genes in porcine granulosa cells in vitro was investigated to explore the role of PLC in porcine granulosa cells.

The apoptosis of granulosa cells is mainly regulated by the caspase-dependent signaling pathways, and most of the apoptosis leads to the terminal differentiation at the antral follicle surface in granulosa cells [3]. Mitochondria are involved in mammalian cell apoptosis [29]. Previous studies found that the activation of PLC has the effect of anti-apoptosis [30], and the inhibition of PKC increases apoptosis [17], which are consistent with our work. The results in this study were in line with the results from previous research that the inhibition of BCL2 family proteins was accompanied by the promotion of BAX and BAK which might be regulated by cell death signals [31, 32], and the activation of BAX, which might regulate downstream genes including caspase 3, could induce apoptosis [33]. An increased abundance of *BCL2* was reported to be accompanied by an increase in *NFκB* expression rather than *BAX* expression [34], and the expression of *BCL2* was the opposite to *BAX* mRNA expression [35], which was the same in our results. In this study, we can speculate that apoptosis of porcine granulosa cells was induced by the supplementation of U73122 at each time point, and mainly inhibited by the m-3M3FBS treatment after 8 h. Annexin/PI binding could indicate that apoptosis and necrosis increase with atresia progression [36].

The PLC enzymes are responsible for hydrolysis of phosphatidylinositol-4, 5- bisphosphate (PIP_2_), an inner membrane component which could produce the second messenger IP3 and DAG, and then released the intracellular Ca^2+^ [37]. The effect of inhibitor U73122 or m-3M3FBS on PLC mRNA and protein expression was affected by both the culture time and their concentration of treatment. The activation of PLC β could increase intracellular Ca^2+^ [38], which is in line with this study that PLC activator increased the intracellular Ca^2+^ concentration and PLC inhibitor decreased the intracellular Ca^2+^ concentration. The PKC activator PLC could be activated by prostaglandin f (2alpha) in the ovary [39]. It was reported that the Wnt/Ca^2+^ signaling pathway could reduce cisplatin resistance, and CaMKII might be an underlying therapeutic target in chemoresistant ovarian cancers [40]. Caln, NFAT1 and NFAT2 are essential to the tumorigenic and metastatic properties of tumor cells in mice, a phenotype which coincides with increased apoptosis in vivo [41]. In this study, the results showed that PLC mediated the abundance of three Ca^2+^-sensitive proteins and affected apoptosis.

We found that *CDC42* mRNA expression was down-regulated by the PLC inhibitor U73122 and up-regulated by the PLC activator m-3M3FBS in porcine granulosa cells, which indicated that CDC42 gene was a target of PLC. The CDC42-PAKs-ERK1/2 MAPK signaling cascade in the prehierarchical follicles of the chicken ovary could mediate the suppression of granulosa cell proliferation, differentiation and follicle selection [42]. In this study, the expression of *NFkB* was inhibited by U73122, while the rate of early apoptosis was increased which was in line with previous studies [43]. The expression of *NLK* mRNA was slightly higher, while β-catenin mRNA expression was decreased with m-3M3FBS supplementation. The result was consistent with previous study [44]. On the other hand, the rapid activation of membranous glucocorticoid receptor (mbGR)/PLC/PKC further lead to the activation of β-catenin [45]. It is possible that different cell types have different regulation mechanisms, and the relationship between PLC and β-catenin still needs further study.

Taken together, these result proved that PLC acts in porcine granulosa cells via several target proteins and genes, and participates in the regulation of apoptosis, which may provide new information to understand the role of PLC in porcine granulosa cells. The regulation of PLC signaling might be instructive to protect the function of mammal ovaries and monitor the drug action in clinical application. Future studies might focus on the effects of the specific mechanism of the regulation.

## Conclusion

Findings of the present study pointed out that PLC played a role in inhibiting the apoptosis rate in porcine granulosa cells. The intracellular Ca^2+^ concentration was declined treated with the addition of 0.5 μM U73122 for 4 h, and increased cultured with 0.5 μM m-3M3FBS for 4 h in porcine granulosa cells. Three Ca^2+^ sensitive proteins (protein kinase C β, calmodulin-dependent protein kinase II α, and calcineurinA) and several downstream genes (*CDC42*, *NFATc1* and *NFκB*) in PLC signaling was positively regulated by PLCB1, and CTNNB was negatively regulated by PLCB1 in porcine granulosa cells.

## Supplementary information

**Additional file 1: Figure S1.** Effect of U73122 on the mRNA abundance of PLCB1 in porcine granulosa cells. Cells were challenged with the doses of U73122(from 0 μM to 5 μM) for the times given. Data are mean ± S.E.M.of three independent replicates. For each treatment, means without common letters are significantly different(*p*<0.05). **Figure S2.** Effect of m3M3FBS on the mRNA abundance of PLC in porcine granulosa cells. Cells were challenged with the doses of m3M3FBS (from 0 μM to 50 μM) for the times given. Data are mean ± S.E.M.of three independent replicates. For each treatment, means without common letters are significantly different(*p*<0.05).**Figure S3.** One representative scatter diagram at each time point for apoptosis induced by U73122 measured with annexin V/PI staining in porcine granulosa cells. Cells were challenged with 0.5 μM U73122 for 4 h (gene) or for the times given(percentage of apoptotic cells), which were processed for annexin V/PI staining and measured by flow cytometry assay. **Figure S4.** One representative scatter diagram at each time point for apoptosis induced by m3M3FBS measured with annexin V/PI staining in porcine granulosa cells. Cells were challenged with 0.5 μM m3M3FBS for 4 h(gene) or for the times given(percentage of apoptotic cells), which were processed for annexin V/PI staining and measured by flow cytometry assay. **Figure S5.** Fluorescence intensity of intracellular Ca^2+^ in porcine granulosa cells. The area in the histogram represents the fluorescence intensity variation of granulosa cells measured by Flow cytometry and analyzed by FlowJo V10. A and B indicate the fluorescence intensity treated with DMF (Control) and U73122; C and D indicate the fluorescence intensity cultured with DMSO (Control) and m-3M3FBS.

## Data Availability

All publicly data generated or analyzed during this study are included in this published article. The data used to support the findings of this study are available from the corresponding author upon request.
