# Supplementary material for: Phospholipase C inhibits apoptosis of porcine primary granulosa cells cultured in vitro
Source: J Ovarian Res. 2019 Sep 25;12:90. doi: 10.1186/s13048-019-0567-4 (PMC6761717; doi:10.1186/s13048-019-0567-4)

**Phospholipase C inhibits apoptosis of porcine primary granulosa cells cultured *in vitro***

**Journal of Ovarian Research**

**HuaLi Chen^a^, YouFu Yang^a^, YouLin Wang^a^, Yuan Li^a^, YaMei He^a，b^, JiaXin Duan^a^, DeJun Xu^a^, YiFei Pei^a^, JianYong Cheng^a^, Li Yang^a,c^, RongMao Hua^a^, XiaoYa Li^a^, Jie Wang^a^, XiaoHan Jiang^a^, HuanShan He^a^, Lin Wu^a^, DingBang Liu^a^ and QingWang Li^a*^**

^a^College of Animal Science and Technology, Northwest A&F University, Yangling, Shaanxi 712100, PR China

^b^Hanzhong Vocational and Technical College, Hanzhong, Shaanxi 723000, China

^c^First Affiliated Hospital of Jiamusi University, Jiamusi, Heilongjiang 154007, China

***Correspondence:** liqingwangysu@hotmail.com

**Figure S1.**Effect of U73122 on the mRNA abundance of PLCB1 in porcine granulosa cells. Cells were challenged with the doses of U73122(from 0μM to 5μM )for the times given. Data are mean±S.E.M.of three independent replicates. For each treatment,means without common letters are significantly different(*p*＜0.05).

**Figure S2.**Effect of m3M3FBS on the mRNA abundance of PLC in porcine granulosa cells. Cells were challenged with the doses of m3M3FBS (from 0μM to 50μM )for the times given. Data are mean±S.E.M.of three independent replicates. For each treatment,means without common letters are significantly different(*p*＜0.05).

**Figure S3.** One representative scatter diagram at each time point for apoptosis induced by U73122 measured with annexin V/PI staining in porcine granulosa cells. Cells were challenged with 0.5 μM U73122 for 4h (gene) or for the times given(percentage of apoptotic cells), which were processed for annexin V/PI staining and measured by flow cytometry assay.

**Figure S4.** One representative scatter diagram at each time point for apoptosis induced by m3M3FBS measured with annexin V/PI staining in porcine granulosa cells. Cells were challenged with 0.5 μM m3M3FBS for 4h(gene) or for the times given(percentage of apoptotic cells), which were processed for annexin V/PI staining and measured by flow cytometry assay.

**Figure S5. Fluorescence intensity of intracellular Ca^2+^ in porcine granulosa cells.** The area in the histogram represents the fluorescence intensity variation of granulosa cells measured by Flow cytometry and analyzed by FlowJo V10. A and B indicate the fluorescence intensity treated with DMF (Control) and U73122; C and D indicate the fluorescence intensity cultured with DMSO (Control) and m-3M3FBS.

**Figure S1**


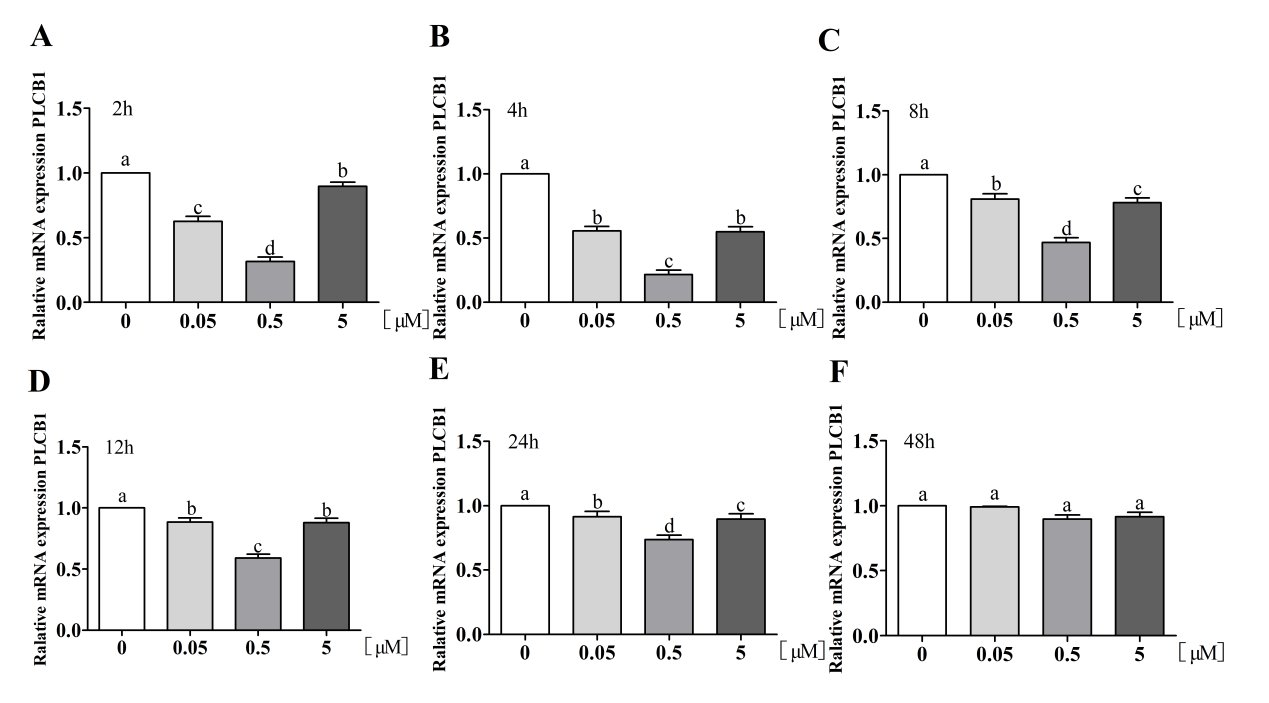


**Figure S2**


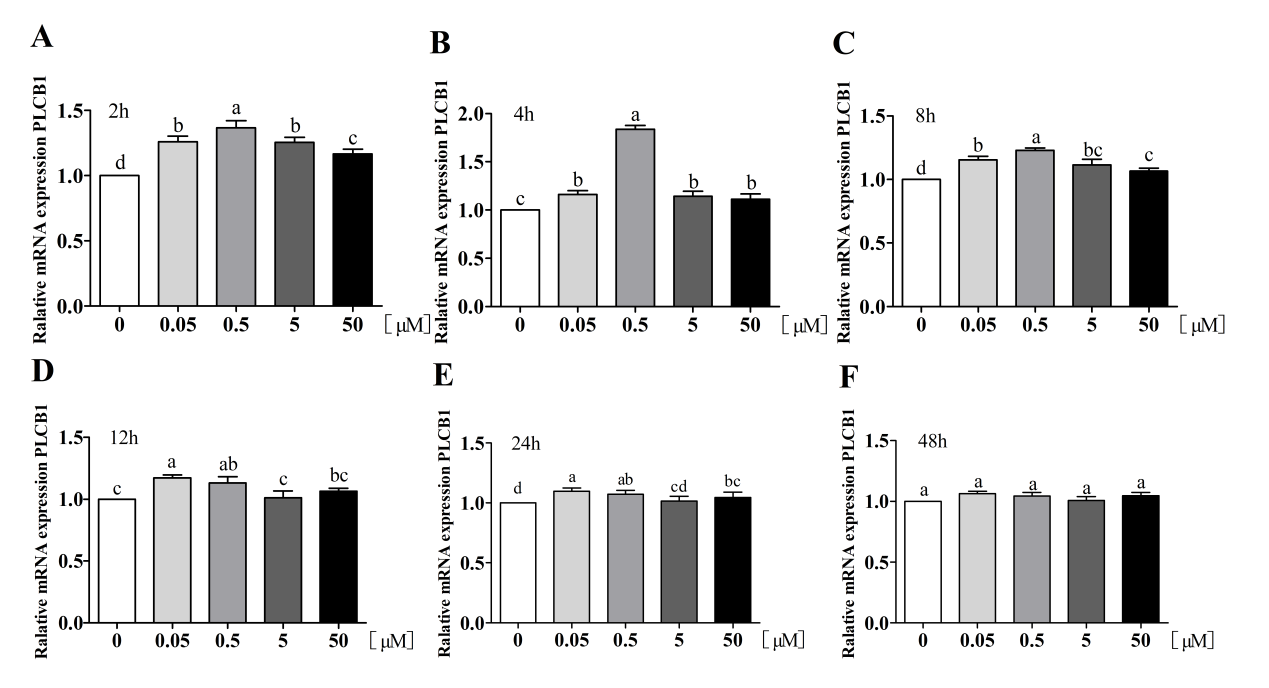


**Figure S3**


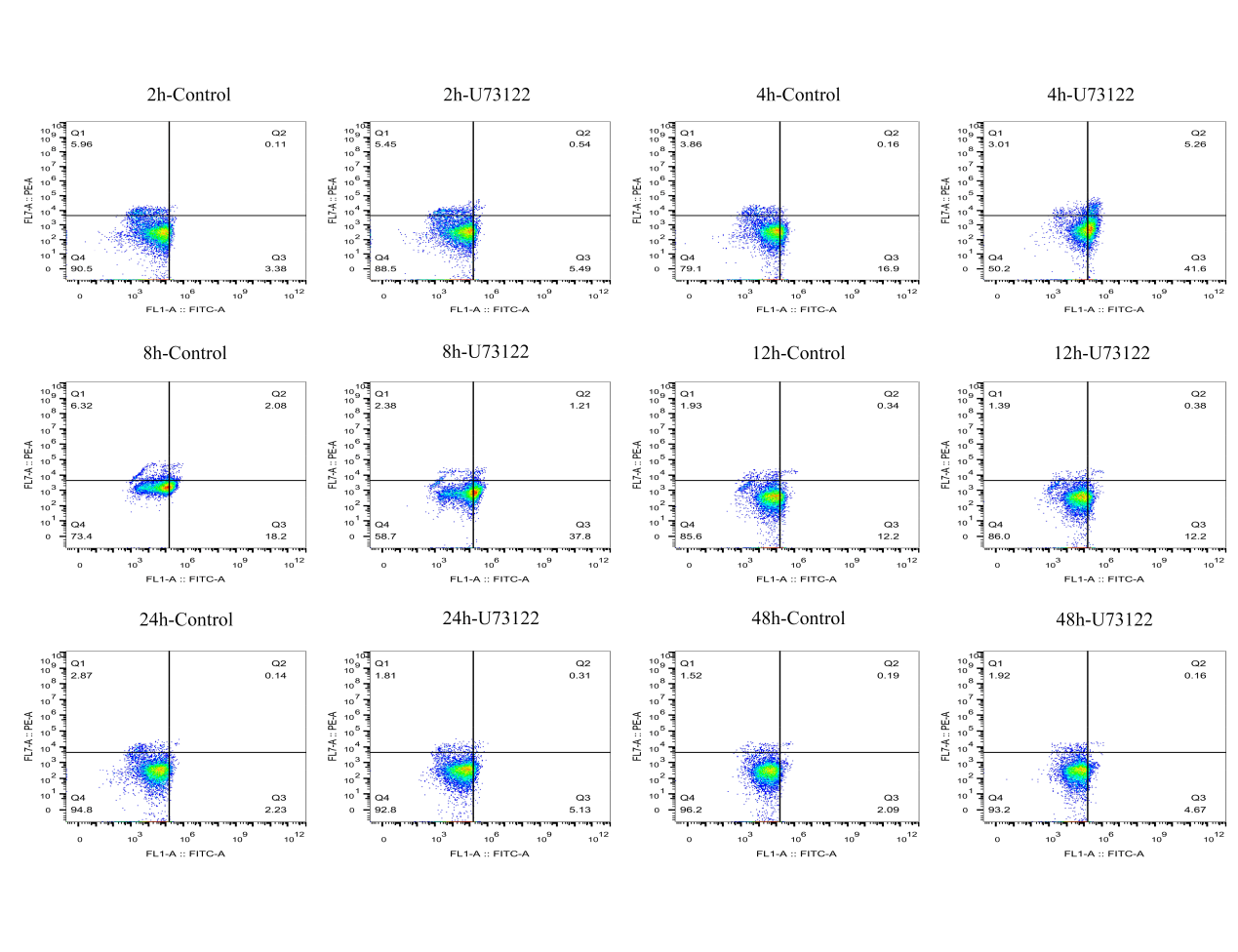


**Figure S4**


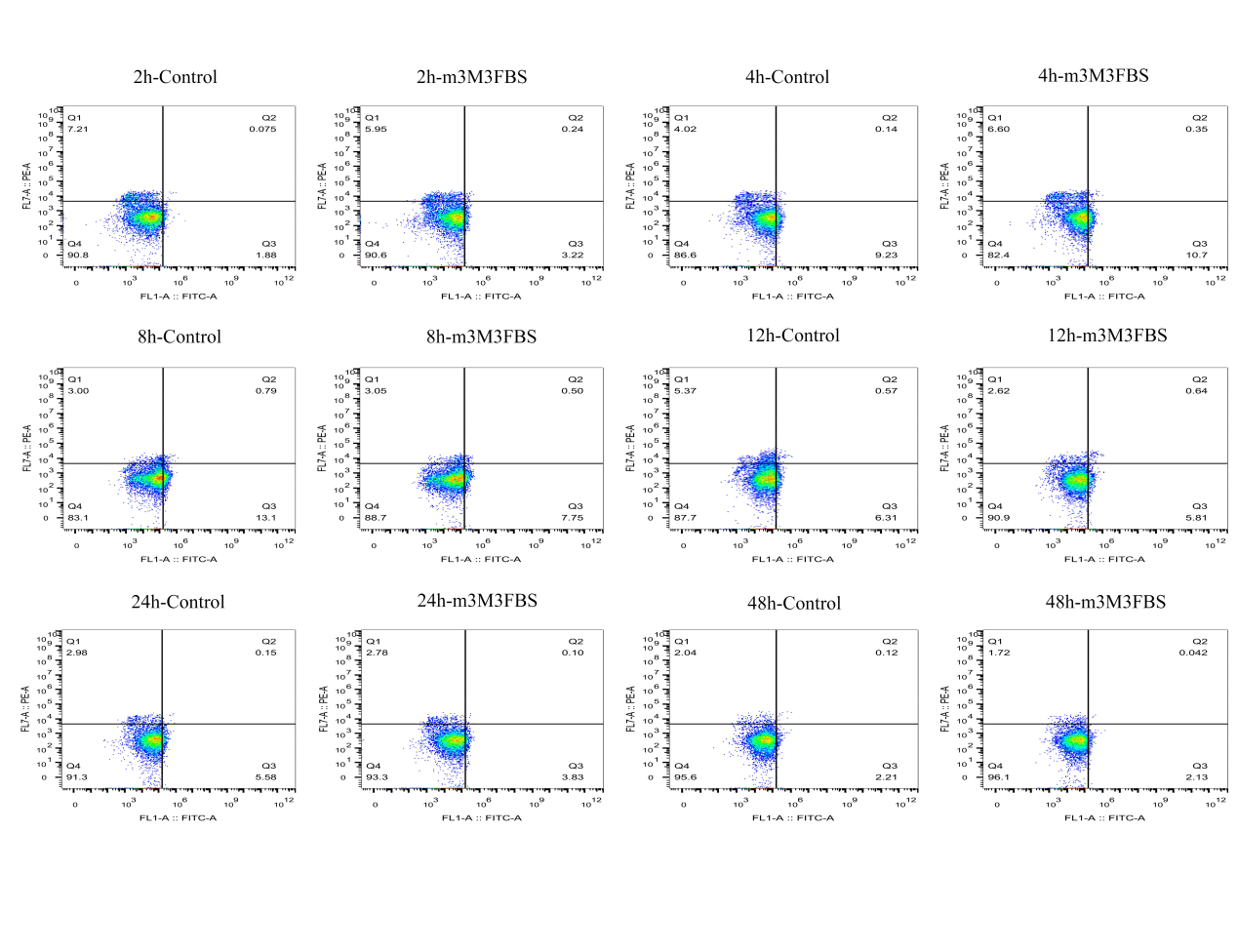


**Figure** S5


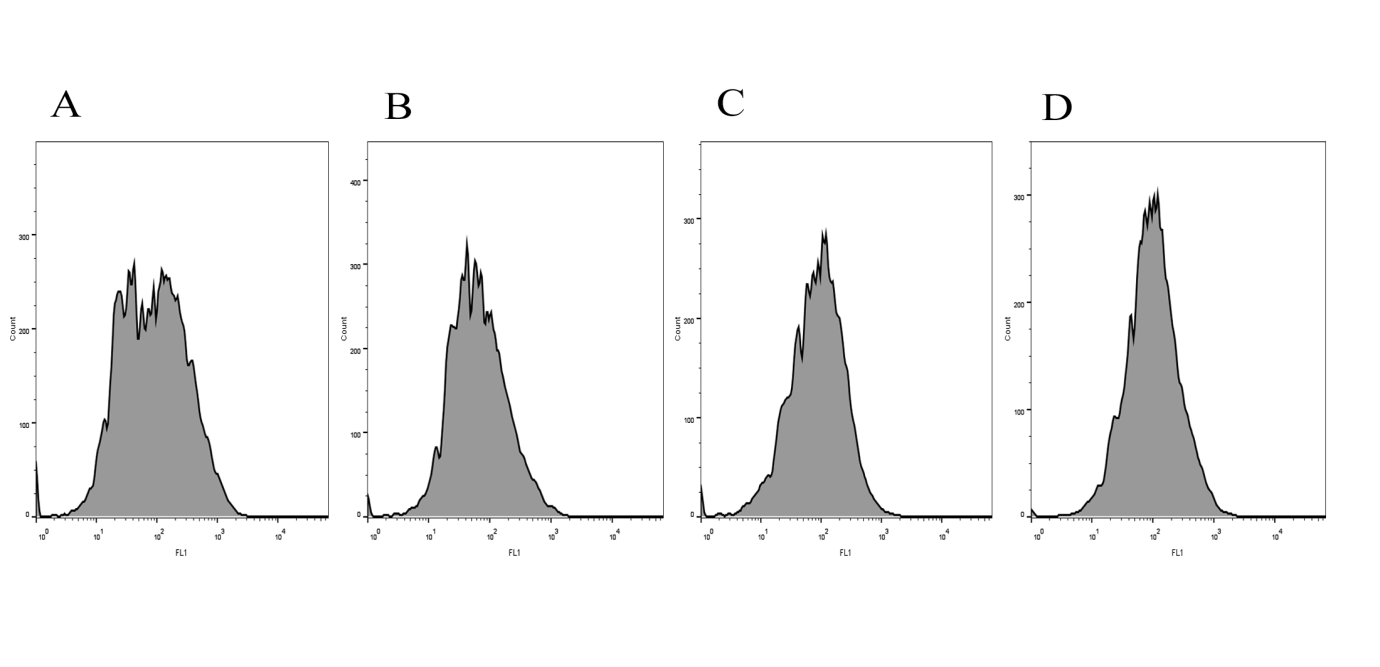

Supplement: Supplementary file 1 — Additional file 1: Figure S1. Effect of U73122 on the mRNA abundance of PLCB1 in porcine granulosa cells. Cells were challenged with the doses of U73122(from 0 μM to 5 μM) for the times given. Data are mean ± S.E.M.of three independent replicates. For each treatment, means without common letters are significantly different(p<0.05). Figure S2. Effect of m3M3FBS on the mRNA abundance of PLC in porcine granulosa cells. Cells were challenged with the doses of m3M3FBS (from 0 μM to 50 μM) for the times given. Data are mean ± S.E.M.of three independent replicates. For each treatment, means without common letters are significantly different(p<0.05).Figure S3. One representative scatter diagram at each time point for apoptosis induced by U73122 measured with annexin V/PI staining in porcine granulosa cells. Cells were challenged with 0.5 μM U73122 for 4 h (gene) or for the times given(percentage of apoptotic cells), which were processed for annexin V/PI staining and measured by flow cytometry assay. Figure S4. One representative scatter diagram at each time point for apoptosis induced by m3M3FBS measured with annexin V/PI staining in porcine granulosa cells. Cells were challenged with 0.5 μM m3M3FBS for 4 h(gene) or for the times given(percentage of apoptotic cells), which were processed for annexin V/PI staining and measured by flow cytometry assay. Figure S5. Fluorescence intensity of intracellular Ca2+ in porcine granulosa cells. The area in the histogram represents the fluorescence intensity variation of granulosa cells measured by Flow cytometry and analyzed by FlowJo V10. A and B indicate the fluorescence intensity treated with DMF (Control) and U73122; C and D indicate the fluorescence intensity cultured with DMSO (Control) and m-3M3FBS. [file 13048_2019_567_MOESM1_ESM.docx]
